# De novo production of six key grape aroma monoterpenes by a geraniol synthase-engineered *S. cerevisiae* wine strain

**DOI:** 10.1186/s12934-015-0306-5

**Published:** 2015-09-16

**Authors:** Ester Pardo, Juan Rico, José Vicente Gil, Margarita Orejas

**Affiliations:** Instituto de Agroquímica y Tecnología de Alimentos, Consejo Superior de Investigaciones Científicas (IATA-CSIC), Agustín Escardino 7, 46980 Paterna, Valencia Spain; Departamento de Medicina Preventiva y Salud Pública, Ciencias de la Alimentación, Bromatología, Toxicología y Medicina Legal, Facultad de Farmacia, Universidad de Valencia, Vicente Andrés Estellés s/n, 46100 Burjassot, Valencia Spain

**Keywords:** Geraniol, Geraniol synthase, Metabolic engineering, Monoterpenes, *Saccharomyces cerevisiae*, Monoterpene bioconversion, Wine aroma, Self-aromatizing wine yeasts

## Abstract

**Background:**

Monoterpenes are important contributors to grape and wine aroma. Moreover, certain monoterpenes have been shown to display health benefits with antimicrobial, anti-inflammatory, anticancer or hypotensive properties amongst others. The aim of this study was to construct self-aromatizing wine yeasts to overproduce de novo these plant metabolites in wines.

**Results:**

Expression of the *Ocimum basilicum* (sweet basil) geraniol synthase (*GES*) gene in a *Saccharomyces cerevisiae* wine strain substantially changed the terpene profile of wine produced from a non-aromatic grape variety. Under microvinification conditions, and without compromising other fermentative traits, the recombinant yeast excreted geraniol de novo at an amount (~750 μg/L) well exceeding (>10-fold) its threshold for olfactory perception and also exceeding the quantities present in wines obtained from highly aromatic Muscat grapes. Interestingly, geraniol was further metabolized by yeast enzymes to additional monoterpenes and esters: citronellol, linalool, nerol, citronellyl acetate and geranyl acetate, resulting in a total monoterpene concentration (~1,558 μg/L) 230-fold greater than that of the control. We also found that monoterpene profiles of wines derived from mixed fermentations were found to be determined by the composition of the initial yeast inocula suggesting the feasibility of producing ‘à la carte’ wines having predetermined monoterpene contents.

**Conclusions:**

Geraniol synthase-engineered yeasts demonstrate potential in the development of monoterpene enhanced wines.

**Electronic supplementary material:**

The online version of this article (doi:10.1186/s12934-015-0306-5) contains supplementary material, which is available to authorized users.

## Background

Aroma is one of the most appreciated traits in assessing wine quality, and among the hundreds of volatile compounds characterized only a small number influence its sensory perception (see [1, 2] and references therein). These aroma active compounds (e.g. terpenes, esters, alcohols) have their origins in grapes, the metabolism of microorganisms (especially the wine-making yeast *Saccharomyces cerevisiae*), and wine aging and storage conditions.

Monoterpenes (a C_10_ class of terpenes mainly derived from grapes) are key odorants associated with the varietal (or primary) aromas of certain white wines. Linalool, geraniol, nerol, citronellol and α-terpineol are the major constituents of aromatic grape varieties (*e.g.* Muscat d’Alexandrie, Gewürztraminer, Riesling), imparting floral and fruity attributes (reviewed in [3, 4]), and certain dietary monoterpenes are of nutraceutical importance because of their antimicrobial, antiviral, anti-proliferative, antioxidative, anxiolytic, hypotensive or anti-inflammatory properties, among other activities (see [5–8] and references therein). Apart from the natural properties of a grape variety, monoterpene content is also influenced by uncontrollable factors such as climate and soil. A large proportion of these monoterpenes is present in grape musts as non-volatile odorless sugar glycoconjugates that can be released enzymatically using industrial glycosidase cocktails or recombinant wine yeast strains expressing such activities (for reviews see [9–11]). Nevertheless, a number of grape varieties are aromatically ‘neutral’ and almost completely lack free monoterpenes and their precursors [4]. Thus there is considerable variability in monoterpene contents in grapes.

Monoterpene biosynthesis in plants is effected by monoterpene synthases (MTPSs). Many of their corresponding genes have been characterized [12, 13] and considerable expansion of these has been observed in grapevine (*Vitis vinifera*) [14, 15]. *S. cerevisiae* wine strains themselves produce only tiny quantities of monoterpenes (e.g. up to 1.2 or 4 μg/L of geraniol and linalool, respectively) [16] because they lack MTPSs and cannot therefore contribute to ameliorating monoterpene deficiency in grape must. Notwithstanding the non-acceptability of GMOs, especially by European wine consumers and industries, vinification by engineered monoterpene-producing wine yeast strains could thus constitute a means to enhance varietal wine aroma. In this regard, successful expression of the *Clarkia breweri* S-linalool synthase (*LIS*) gene in a *S. cerevisiae* wine yeast strain has provided proof of concept by virtue of de novo production of linalool in wines to about 19 μg/L [17]. This metabolic manipulation was possible because plant MTPSs catalyze the synthesis of monoterpenes from geranyl pyrophosphate (GPP) in a single step, and *S. cerevisiae* has enough free GPP (an intermediate in ergosterol biosynthesis) under vinification conditions to be used as a substrate by these plant enzymes. In addition *S. cerevisiae* has the ability to metabolize supplemented monoterpenes, the bioconversions of (i) geraniol into citronellol, linalool, nerol and geranyl acetate, (ii) nerol into geraniol, linalool and α-terpineol, (iii) linalool into α-terpineol and (iv) citronellol into citronellyl acetate having been reported (see [18–20] and references therein). Thus an engineered monoterpene-producing yeast could also play a valuable additional role in the development of wine aroma by producing a wider spectrum of monoterpenes.

Previous work has shown that wine yeast strain T_73_ has a greater inherent capacity for recombinant monoterpene production compared to other laboratory and industrial wine strains [21]. Here we report the substantial modification of the terpene profile of a wine produced from a neutral grape variety using the T_73_ strain expressing the geraniol synthase (*GES*) gene from *Ocimum basilicum* (sweet basil) [22].

## Results and discussion

### Production of geraniol by a wine yeast strain expressing the *GES* gene of *O. basilicum* and its metabolic fate in synthetic defined (YPD) media

**Fig. 1:**
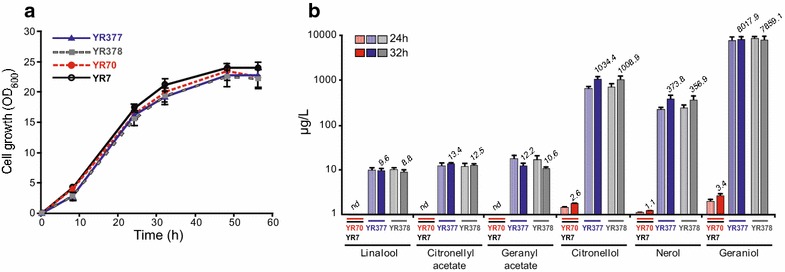
Growth and monoterpene production in YPD of recombinant wine yeast T_73_-4 expressing the *O. basilicum* *GES* gene. **a** Growth curves of T_73_Ges (YR377 and YR378) and control strains YR70 (T_73_-4 transformed with the empty plasmid) and T_73_. **b** Monoterpene production at 24 and 32 h by YR377, YR378 and controls.* Numbers* above the* bars* corresponding to 32 h indicate μg/L. Terpene concentrations are represented on a logarithmic scale. Results are presented as the mean and standard deviations of two independent assays with three replicates each.

The truncated *O. basilicum GES* cDNA [22] (GenBank Accession No. AY362553) coding for a geraniol synthase lacking the first 34 codons—which encode the plastid transit peptide—was cloned under the control of the *S. cerevisiaeACT1* (encoding actin) promoter (*ACT1*_p_) and the *HIS3* (encoding imidazole glycerol-phosphate dehydratase) terminator (*HIS3*_t_) into the binary vector YEplac195 [23]. The resulting plasmid (YEp195Ges) was used to transform the *S. cerevisiae* T_73_-4 [24] wine strain and the uracil prototrophic (ura^+^) transformants YR377 and YR378 (T_73_Ges) were isolated. The growth rates of YR377, YR378 and the control strain YR70 (T_73_-4 transformed with the empty plasmid) on liquid YPD media were almost identical, albeit slightly slower than that of the industrial strain T_73_ (Fig. 1a) as observed previously for other recombinant yeasts [17]. This indicates that neither the amount of geraniol nor the putative reduction of precursors from the isoprenoid pathway apparently produce deleterious effects on yeast growth under such conditions. In addition, GC and GC–MS analyses of these culture media showed similar extraordinarily high geraniol yields (8,017.85 ± 1,245.81 and 7,859.12 ± 1,614.62 μg/L after 32 h) (Fig. 1b). These levels are about 16-fold higher than those produced by recombinant *S. cerevisiae* laboratory strains expressing the same *GES* gene, about 1.6-fold the amount produced by laboratory yeasts co-expressing *GES* and an optimized farnesyl diphosphate synthase [25, 26], and about 120-fold the amount of linalool excreted by engineered T_73_-4 wine strains expressing *LIS* [17, 21]. These results clearly reinforce the previously shown importance of the genetic background of this industrial yeast for monoterpene production [21] but also that of the monoterpene synthase being expressed. In contrast to the T_73_Lis strains that produced linalool as the only end product, and in agreement with the reported ability of *S. cerevisiae* T_73_ to metabolise supplemented geraniol and its reaction products [20], the T_73_Ges strains produced geraniol (84.83%) and geraniol derivatives i.e. citronellol (10.92%), nerol (3.90%), linalyl acetate (0.13%), geranyl acetate (0.12%) and linalool (0.1%). As expected, monoterpene production by the control strains lacking *GES* (YR70 and T_73_) was practically negligible (7.13 ± 1.12 μg/L; >1,300-fold lower than YR377 and YR378) (Fig. 1b). YR377 was chosen for the microvinification experiments.

### Aromatic wines from neutral grapes using the self-aromatizing wine yeast YR377

**Fig. 2:**
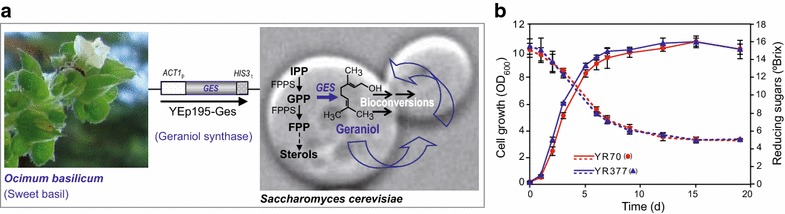
Analyses of microvinifications. Microvinifications were carried out with YR377 (T_73_Ges) and the YR70 control strain transformed with the empty vector. **a** Schematic representation of the engineered mevalonate pathway in the T_73_Ges strains. IPP, isopentenyl pyrophosphate; GPP, geranyl pyrophosphate; FPP, farnesyl pyrophosphate; FPPS, FPP synthase. **b** Growth curves and kinetics of sugar consumption by YR377 and YR70 during the course of fermentations. Results are presented as the mean and standard deviation.

Microvinification experiments were carried out in parallel on sterile Parellada white grape must using the wine yeast strain T_73_-4 that carries the *GES* expression cassette (YR377) and a control strain lacking *GES* (YR70). Both alcoholic fermentations progressed similarly (Fig. 2b) and reached completion in about 19 days leaving approximately 2 g/L residual sugar (i.e. dry wine). Given the persistence of the ura^+^ phenotype (around 85%) in YR377 and hence high maintenance of the *GES* expression cassette throughout the process it is evident that neither the expression of the *GES* gene nor its consequences affected the growth or fermentative capacity of the engineered wine strain.

**Fig. 3:**
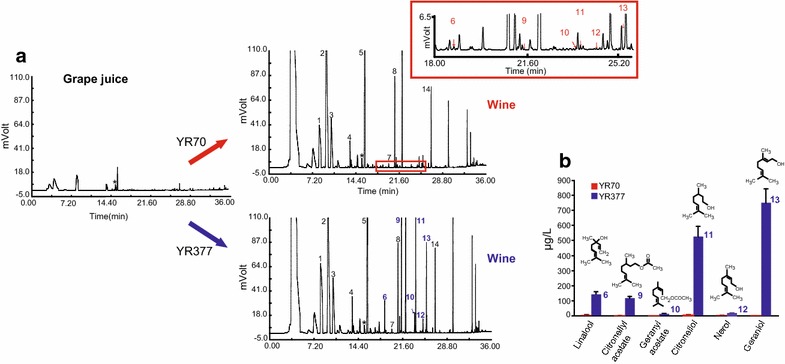
Presence of geraniol, citronellol, linalool, nerol, citronellyl acetate and geranyl acetate in wines produced by YR377. **a** Comparison of the chromatograms of wines produced by YR377 (T_73_Ges) and YR70 (control). *Arrows* indicate peaks of geraniol and its derivatives.* Peak numbers* refer to the aromatic compounds listed in Table 1. *Asterisks* indicate retention time of the internal control 2-octanol. The insert amplifies the region of the chromatogram corresponding to the monoterpenes. **b** Geraniol, and geraniol derivative structures and their contents in wines.

To evaluate the influence of *GES* expression on wine aroma, volatile profiles were determined by GC and GC–MS (Fig. 3a). As expected given the aromatic neutrality of the Parellada grape, free geraniol was undetectable in wines produced by YR70. In contrast, geraniol concentrations (~750 μg/L) well in excess of its olfactory perception threshold (40–75 μg/L) and exceeding those present in wines obtained from the highly aromatic Muscat grapes (Additional file 1: Table S1) were found in wines fermented with the ‘self-aromatizing’ wine yeast YR377 (Fig. 3b; Table 1). Remarkably, GC analysis (Fig. 3) showed that apart from the geraniol peak there were also notable quantities (~810 μg/L) of additional monoterpenes and esters associated with strain YR377: citronellol, linalool, nerol, citronellyl acetate and geranyl acetate, resulting in a total terpene concentration >220-fold greater than the control wine. With the exception of nerol and citronellyl acetate, the other compounds are present above their perception thresholds (Table 1). The presence of geraniol and its derivatives will enrich these wines with flowery and fruity notes.

**Table 1:** Concentrations (μg/L), odor quality and thresholds of a selected subset of aromatic compounds found in Parellada wines fermented with the T_73_Ges strain

Compound^a^	Aroma descriptor^b^/OTVs^c^ (μg/L)	YR70^d^	YR377^d^
*Monoterpenes*			
Linalool (6)	Floral/(6)	2.36 ± 0.41	141.18 ± 19.55
Citronellyl acetate (9)	Fresh, fruity reminiscent of rose/(250)	0.81 ± 0.14	116.32 ± 12.23
Geranyl acetate (10)	Pleasant, flowery reminiscent to rose lavender/(9)	nd	12.84 ± 1.51
Citronellol (11)	Rose-like/(40)	2.27 ± 0.38	526.35 ± 67.17
Nerol (12)	Fresh, sweet, rose-like/(300)	1.33 ± 0.06	12.62 ± 1.03
Geraniol (13)	Rose-like/(40–75)	nd	749.17 ± 93.64
α-Terpineol	Lilac/(330–350)	nd	nd
*Alcohols*			
3-Methyl 1 butanol (2)	Fusel oil, whiskey characteristic pungent/(250–300)	79,376.67 ± 12,716.18	81,726.68 ± 4,294.14
1-Hexanol (4)	Herbaceous, woody, sweet, green fruity/(2,500)	343.79 ± 93.98	383.42 ± 54.91
1-Octanol (7)	Fresh, orange–rose/(110–130)	24.13 ± 4.24	27.21 ± 3.59
2-Phenylethyl alcohol (14)	Rose-like (750–1,100)	24,524.45 ± 3,065.82	26,508.69 ± 2,570.46
*Esters*			
Isoamyl acetate (1)	Fruity, banana, sweet, fragrant/(2)	458.55 ± 49.51	484.51 ± 79.92
Ethyl caproate (3)	Fruity with a pineapple–banana note, winery/(1)	190.52 ± 21.36	175.71 ± 16.52
Ethyl caprylate (5)	Pleasant, fruity, floral, wine-apricot note/(15)	225.23 ± 17.95	178.66 ± 23.60
Ethyl decanoate (8)	Fruity reminiscent of grape (cognac)/(23–122)	61.42 ± 5.50	56.46 ± 6.60
*Total monoterpenes*		6.77 ± 0.99	1,558.48 ± 195.13

nd, not detected.

^a^Numbers between brackets refer to the GC peaks in Fig. 3.

^b^Aroma descriptors according to Feranoli’s [35].

^c^Odor detection threshold values (OTVs) in water (μg/L) were taken from the lists of Leffingwell & Associates (http://www.leffingwell.com/).

^d^Values represent the means and standard deviation of two different microvinifications, three technical replications and two analytical replications.

GES and *E. coli*-expressed recombinant GES both exclusively catalyze the synthesis of geraniol from GPP [22]. Our comparative GC–MS data (Figs. 2, 3) revealed that the same terpenes found in wine were also excreted by YR377 when grown in synthetic (YPD) medium. Thus during vinification the enzymatic activities intrinsic to this wine yeast strain are also able to metabolize geraniol and its derivatives resulting in their conversion to other monoterpenes and aromatic esters, a situation resembling the metabolic diversion occurring in tomato expressing the *GES* gene [27]. The reduction of geraniol to citronellol and the acetylation of geraniol and citronellol are probably catalyzed by the oxidoreductase Oye2 and the alcohol acetyl transferase Atf1 [28], respectively. An obvious strategy to further expand our ability to modulate wine aroma would therefore be to promote or suppress the formation of these geraniol derivatives by modification of these enzyme activities.

To investigate whether *GES* expression could lead to additional changes in a wine’s volatile profile, determinations of other volatile compounds of oenological relevance were carried out on both recombinant yeast derived and control wines. The compositions and concentrations of higher alcohols (e.g. 2-phenylethyl alcohol) and acetate esters (e.g. isoamyl acetate), the presence of which is considered favorable for the aromatic properties of wines, were seen to be statistically similar in wines fermented with YR377 and YR70 strains (Table 1).

Introduction of the *C. breweriLIS* gene into wine yeast strain T_73_-4 (T_73_Lis) under the control of the *TDH3* yeast promoter was our first attempt to construct a self-aromatizing wine yeast [17]. This resulted in de novo accumulation in wine of linalool alone to levels exceeding its odor perception threshold. Remarkably, the amount of geraniol-derived linalool produced by YR377 (T_73_Ges) was about 7.5 times greater than that obtained with T_73_Lis (~141 versus ~19 μg/L) and the total de novo terpene concentration is more than 80-times greater, illustrating the importance of the MTPS employed in engineering strain T_73_. These results justify the strategy of engineering the wine yeast isoprenoid pathway as a means of achieving efficient plant-derived aromatic monoterpene production during alcoholic fermentation.

### Mixed fermentation with T_73_Ges and *S. cerevisiae* strains not producing monoterpenes serves to modulate levels of terpenes

In order to assess whether it would be feasible to produce wines with a predetermined monoterpene content, vinifications were conducted using mixed starters (1:1) of yeast strains YR377 and YR70 and were compared to those obtained using pure cultures of YR377. The monoterpene profiles of wines derived from mixed fermentations were directly related to the composition of the initial inocula. Thus the amounts of geraniol (~388 μg/L) and its derivatives (~311 μg/L) detected were about half of those obtained using inocula of YR377 alone (Table 2).

**Table 2:** Concentrations (μg/L) of geraniol and derivatives found in Parellada wines co-fermented with *GES* strains

Monoterpenes	YR70:YR370^a^
Linalool (6)	57.19 ± 7.76
Citronellyl acetate (9)	40.68 ± 3.32
Geranyl acetate (10)	5.90 ± 1.34
Citronellol (11)	201.28 ± 19.69
Nerol (12)	5.53 ± 0.18
Geraniol (13)	388.35 ± 8.96
*Total*	698.93 ± 41.25

Numbers between brackets refer to the GC peaks in Fig. 3.

^a^Values represent the means and standard deviation of two different microvinifications, three technical replications and two analytical replications.

Terpenes are also important flavor compounds in other fermented drinks. Geraniol, linalool and citronellol have all been shown to be important contributors to the floral, fruity and citrus flavors of beer [29], and biotransformations of these monoterpenes by ale and lager yeasts have been reported [19]. Engineered brewing-yeasts designed as vehicles for the de novo production of these monoterpenes thus have potential for use in the brewing industry. Moreover, certain monoterpenes have been shown to display a plethora of potential health benefits (see [5–8] and references therein).

## Conclusions

**Fig. 4:**
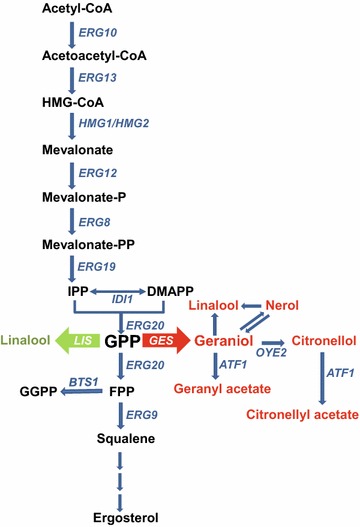
Schematic representation of the isoprenoid pathway in *S. cerevisiae* including the branch point to monoterpenes. Gene names of *S. cerevisiae* appear in* blue*. *Red* and *green arrows* indicate engineered steps to increase monoterpene content in wines (this work and [17], respectively) catalysed by plant linalool (LIS) and *O. basilicum* geraniol (GES) synthases. Monoterpene bioconversions appear with *red letters* [18–20, 28]. HMG-CoA, 3-hydroxy-3-methylglutaryl-coenzyme A; IPP, isopentenyl pyrophosphate; DMAPP, dimethylallyl pyrophosphate; GPP, geranyl pyrophosphate; FPP, farnesyl pyrophosphate; GGPP, geranyl geranylpyrophosphate.

These results demonstrate the considerable potential for geraniol-engineered yeasts in the development of wines with aromas ‘à la carte’. Fermentation of grape musts with these and/or other yeast strains expressing novel plant MTPS genes and thus the possibility of producing monoterpenes absent from grapes will provide variety and novelty to the wine industry. Approaches including the manipulation of enzyme activities responsible for monoterpene bioconversions [28], the engineering of rate-limiting reactions in the mevalonate pathway [21] and/or the possibility of using diverse mixed starters to pre-determine monoterpene content could contribute to the enhancement of complexity in wine aroma (Fig. 4).

The work reported brings to the fore once again the question of whether modern genetic technologies, in this instance for improving wine yeasts, may become acceptable to industry and consumers given the continued resistance to transgenic foods mainly in Europe. The advance reported in our study illustrates the biotechnological improvement of a food beyond the use of this type of technology for generating resistance to herbicides and pests via the genetic manipulation of a plant, and instead offers a clear alternative to transgenic grapes engineered to enhance free monoterpene content.

## Methods

### Strains and culture conditions

*Escherichia coli* DH5α [*endA1*, *hsdR17*, *gyrA96*, *thi*-*1*, *relA1*, *supE44*, *recA1*, Δ*lacU169* (Φ*80 lacZ*Δ*M15*)] was used for cloning experiments and plasmid propagation. *S. cerevisiae* wine strain T_73_-4 [*ura3*::470/*ura3*::470] [24] (derived from T_73_, Lallemand) was used for *GES* expression. *E. coli* was maintained in LB medium (1% tryptone, 0.5% yeast extract, 1% NaCl) with or without 100 μg/mL ampicillin. *S. cerevisiae* strains were maintained in YPD-rich medium (1% yeast extract, 2% bacteriological peptone, 2% glucose) or SD-minimal medium (0.17% yeast nitrogen base without amino acids—Difco Laboratories, Detroit, USA—2% glucose, 0.5% ammonium sulphate) with or without 20 mg/L uracil. For solid media, 1.5% agar was added. To determine terpene yields of recombinant yeasts, aliquots from overnight cultures of selected transformants grown in SD medium lacking uracil were transferred to 250 mL flasks containing 50 mL of YPD medium at an initial OD_600_ of 0.05. Yeast cultures were grown with continuous shaking (200 rpm) at 30°C and aliquots of the cultures were taken at different times.

### Construction of yeast plasmids carrying the *GES* gene of *C. breweri* and yeast transformation

The *GES* cDNA was obtained from pCRT7/CT-TOPO/GES [22] via PCR as a 1.6-kb *EcoR*I [T4 DNA polymerase treated for blunt-ending]-*Bsp*LU11I fragment using the oligonucleotide pair GES-L35-Bs (5′-CCCACGCT*AC****ATGT*CT**GCTTGCACGCCTTTGG-3′; *Bsp*LU11I is in italics and the artificial translation start site codon ATG and the GES-S35 TCT codon appear in bold) and GES-STOP-RI (5′-CCCCC*GAATTC*TATTTATTGAGTGAAGAAGAGG-3′). The *HIS3*_t_ was isolated as a 0.66-kb *Hinc*II-*Sph*I fragment obtained by PCR using genomic DNA of the *S. cerevisiae* strain FY1679 (*MAT***a**/*MAT*α *ura3*-*52*/*ura3*-*52)* and the oligonucleotide pair His3_SalI (5′-AG*GTCGAC*TAGTGACACCGATTATTTAAAGCTG-3′) and His3_SphI (5′-AG*GCATGC*GAATTCGGATCCTCGGGGACACCAAATATGG-3′). These two fragments were subcloned downstream of *ACT1*_p_ in the plasmid YEpACT4 [30] previously digested with *Nco*I and *Sph*I, thereby generating plasmid YEp181Ges (2 μ; *LEU2*). The expression cassette *ACT1*_p_::*GES*::*HIS3*_t_ was isolated from this plasmid as a 2.8-kb *Eco*RI fragment and subcloned into the same site of YEplac195 (2 μ; *URA3*). The resulting plasmid (YEp195Ges) was used to transform the *S. cerevisiae* T_73_-4 [24] wine strain and uracil (ura^+^) prototrophic transformants (T_73_Ges) were thus isolated. To obtain the control strain YR70, T_73_-4 was transformed with YEplac195.

DNA manipulations were performed following standard protocols [31]. PCR fragments were individually cloned into the pGEM-T Easy vector (Promega) and the absence of mutations was confirmed by sequencing. Transformation of the T_73_ derived strain was done using lithium acetate to permeabilize the cells as previously described [24, 32]. Transformants were selected and maintained on SD plates without uracil. For plasmid stability analyses, transformants were grown under both selective (SD) and non-selective (YPD) conditions and the colonies growing under each condition were counted.

### Microvinifications

Two temporally independent microvinifications were done in triplicate at 20°C using 250 mL glass bottles containing 200 mL of Parellada white grape must (Villafranca del Penedés, Spain). The must (ºBrix ~15) was centrifuged and sterilized with 0.2% (v/v) dimethyl dicarbonate (Velcorin; Bayer, Levercusen, Germany) and inoculated with 9 × 10^5^ cells/ml from overnight cultures of YR70 (uracil nutritional control) and YR377 (T_73_Ges). Samples were collected periodically to measure yeast growth and sugar consumption and thus monitor the progress of the fermentations. Sugar concentrations were initially measured as Brix grades using a Euromex RD. 5645 digital refractometer. After 15 days ºBrix stabilized to about 5, and reducing sugar concentrations were measured using the Nelson–Somogyi method [33, 34] to determine the end of the fermentations (‘dry wine’; sugar concentration below 2 g/L). At this point (day 19), the persistence of the plasmids was measured (% colonies grown on selective SD compared to those grown on complete YPD medium), the wines were centrifuged to remove yeast cells and then transferred to new bottles that were kept at −20°C until their analysis.

### GC–MS analysis of volatiles

Geraniol, geraniol derivatives and other volatiles were extracted and analyzed by headspace solid-phase microextraction (HS-SPME) using poly(dimethylsiloxane) (PDMS) fibers (Supelco, USA) coupled to gas chromatography (GC) and GC–mass spectrometry (MS) as previously reported [17]. 2-Octanol (0.2 μg) was used as internal control. Identification of compounds was determined by comparing retention times and mass spectra with those of standards using a Thermo-Scientific model Focus-GC equipped with a HP-Innowax column (length 30 m; inside diameter 0.25 mm; film thickness 0.25 μm) and a Thermo Trace GC Ultra gas chromatograph coupled to a Thermo DSQ mass spectrometer (Thermo-Scientific), under the same chromatographic conditions. Ion spectra of the peaks of interest were identified by comparison with computerized libraries (e.g. Wiley6, NIST). The oven temperature was programmed as follows: 60°C for 5 min, raised to 190°C at 5°C/min, then raised to 250°C at 20°C/min and held 2 min at 250°C. The injector temperature was 220°C. Helium was the carrier gas at 1 mL/min in the splitless mode. Compounds were quantified by integrating the peak areas of GC chromatograms.

## Additional file

Additional file 1:
**Table S1.** Concentrations (μg/L) of fundamental monoterpenes found in wines made from different aromatic and neutral grape cultivars.

## Abbreviations

GC: gas chromatography
GC–MS: gas chromatography–mass spectrometry
GES: geraniol synthase
GPP: geranyl pyrophosphate
LIS: linalool synthase
MTPS: monoterpene synthase
MVA: mevalonic acid
OTV: odour threshold value
