# Supplementary material for: De novo production of six key grape aroma monoterpenes by a geraniol synthase-engineered S. cerevisiae wine strain
Source: Microb Cell Fact. 2015 Sep 16;14:136. doi: 10.1186/s12934-015-0306-5 (PMC4574175; doi:10.1186/s12934-015-0306-5)
Supplement: Additional file 1: — Table S1. Concentrations (μg/L) of fundamental monoterpenes found in wines made from different aromatic and neutral grape cultivars. [file 12934_2015_306_MOESM1_ESM.doc]

**Additional Table S1**

**Concentrations (μg/L) of fundamental monoterpenes found in wines made from different aromatic and neutral grape cultivars**

| **Grape variety** | **Linalool** | **Citronellol** | **Nerol** | **Geraniol** | **α-Terpineol** | **Source** |
| --- | --- | --- | --- | --- | --- | --- |
| Muscat of Alexandria | 455 | nd | 94 | 506 | 78 | [1] |
| Muscat of Frontignan  Muscat of Bornova  Muscat of Pantelleria  Muscat of Siracusa  Muscat of Noto  Gewürztraminer | 473  179  592-801  1099  2942  6  6-190 | nd  58  3-43  11  15  12  nd | 135  nr  8-16  8  49  43  Tr-20 | 327  171  13-22  4  48  218  20-70 | 87  140  nr  nr  nr  3  30-35 | [1]  [2]  [3]  [3]  [3]  [1]  [4] |
| Riesling  Scheurebe  Albariño  Sauvignon Blanc  Listan  Parellada | 103  175  189  40  185  8-93  307  80  1-3  17  20  5  1-2 | 42  42  31  4  8  1-5  15  nd  1-3  2  7  2  0-2 | <26  nr  82  23  >26  3-43  nr  97  1-2  5  >26  1  0-2 | 179  221  nr  35  46  14-187  38  58  13-24  5  13  11  10 | 65  nr  37  25  128  7-52  nr  37  1-4  9  16  1  0-1 | [5]  [6]  [7]  [1]  [5]  [8]  [6]  [1]  [9]  [1]  [5]  [9]  [9] |

nd: not detected; nr: not reported; Tr: Traces

**Additional References**

1. Ribéreau-Gayon P, Glories Y, Maujean A, Dubourdieu D: *Handbook of enology, volume 2, The chemistry of wine*. John Wiley & Sons, Ltd., Chichester, West Sussex, UK, 2006.

2. Selli S, Canbas A, Cabaroglu T, Erten H, Günata Z: **Aroma components of cv. Muscat of Bornova wines and influence of skin contact treatment**. *Z. Food Chem* 2006, **94**:319-236.

3. Barbera D, Avellone G, Filizzolam F, Montem L, et al: **Determination of terpene alcohols in Sicilian Muscat wines by HS-SPME-GC-MS**. *Nat Prod Res* 2013, **27**:541-547.

4. Bakker J, Clarke RL: *Wine Flavour Chemistry*. Wiley-Blackwell, Chichester, UK, 2012.

5. Mikulíková R, Goliás J, Mrázová V: **SPME-GC-MS analysis of volatile compounds in Czech white wines from five grape varieties**. *Mitt Klosterneuburg* 2009, **59**:159-165.

6. Guth H: **Quantitation and sensory studies of character impact odorants of different white wine varieties**. *J Agric Food Chem* 1997, **45**:3027-3032.

7. Vilanova M, Genisheva Z, Graña M, Oliveira JM: **Determination of odorants in varietal wines from international grape cultivars (*Vitis vinifera*) grown in NW Spain**. S Afr J Enol Vitic 2013, **34**:212-222.

8. Marais J: **Terpenes in the aroma of grapes and wines: a review**. *S. Afr J Enol Vitic* 1983, **4**:49-58.

9. López-Tamames E, Carro-Mariño N, Günata YZ, Sapis C, Baumes R, Bayonove C: **Potential aroma in several varieties of Spanish grapes**. *J Agr Food Chem* 1997, **45**:1729-1735.
